# Where Shall We Recreate and How?

**Published:** 1880-07

**Authors:** Ben. H. Pratt


// THE BISTOURY.

Vol. XVI. ELMIRA, N. Y., JULY, 1880. No. 2.

### Contributions.

### WHERE SHALL WE RECREATE, AND HOW?

BEN. H. PRATT.

When the sizzling days come, and we steam and sweat and frizzle and fry—when there is comfort nowhere and discomfort everywhere, we begin to fret. It is not good for man to fret. Fretting wears out more men than labor does, be the labor what it may, whether of head or arm. These last days are provocative of fretting. Clothes become a burden. We fret our clothing down to a minimum and then wish that the minimun were zero. We would like to shed our clothes and sit in the only garb that nature has given us. As somebody has said, we'd like to divest ourselves of our flesh and sit in our bones. As somebody else has added, we'd like to go a step further, punch out our marrows and let the zephyrs circulate through our osteal cavities. We suffer because we swelter. We are hot—Oh! so hot! and there is no coolness anywhere.

As I said, hot weather begets discomfort of the keenest sort. Discomfort begets thinking of one's self, and when one begins to settle one's thoughts upon one's self, one grows morbid. Morbidity begets a mental disease, and a diseased mind so quickly communicates its malady to the body, that before one realizes anything else, he realizes that he is all wrong physically. When one gets into that peculiar situation in which he believes himself not well, and yet doesn't know what is the matter with himself, he is in a bad fix. Something must be done. There's no end to his imaginings as to what must be done, especially if he is absolutely certain that he doesn't know what must be done, and nobody seeks to advise him as to what he must do. One is in a bad way when one has made up his mind, to a dead certainty, that he isn't a well man. There is but one thing that will help one out of this fix—change. He must have a change of air, of climate, of scene, of occupation. He concludes that he is run down and wants winding up—that he's overworked and needs rest—that he can't stand it any longer unless he can get away—he must go away. Once settled upon this point he is easier immediately. Even if he isn't so sure that change of place, and air, and scene, and occupation will do him any real good, it will, at least, cause him to forget himself for a time in being compelled to give his attention to something besides himself. He makes up his mind—he gets ready to go somewhere—poverty can't keep him at home—business can't hold him—duty can't detain him—everything must subserve his single purpose, and he must go; come what may, going is the only thing that he thinks of—his mind is made up definitely, unchangably.

The question now arises, "Where shall we go?" We don't want to go where we'll do, and see, and hear, and feel, and smell, and eat the same sort of things we do, see, hear, feel, smell and eat at home; otherwise where's the good of going away. We want change—not a little change from our usual life, but an entire change, if such be possible. We want to leave the old hum-drum life at home, and discard everything connected with it. We want to drop it right where we start from, and bid it a hearty farewell for so long, be the period greater or less, as we remain away. We don't want to do anything the way we did it at home. We don't want to see the same things, nor hear the same sort of noises, nor breathe the same kind of air, nor eat the same kind of food, nor smell any of the same old odors that we're sick of and have run away from. We wan't to recreate, re-create, make ourselves over again, get rid of all the old that's in us, shed all the old material, and get just as much new as we can gather in and stow away during our recreation, our rebuilding, our physical and mental renovation.

Having thus resolved to go away with such a purpose, we are prepared, in a general way, to know about where we ought to go. We want the antipodes of our present. In a general, sweeping way, we say, if we are citizens of a big town, and are in daily contact with our own kind, we want to go where we will see the least possible number of people. If we are country folks, and not much used to mingle with mankind, we want to go where we can see and mix with lots of people ; where we'll see crowds every day and hour. If we live inland, and have always been familiar with hills, and mountains, and woods, and fields, and streams, we want to go to the sea shore and get an entire new range of objects to dwell upon, new sounds to startle us, new everything to arouse our curiosity and set us to wondering and inquiring. The inlander wants ocean, and ships, and beach, and expanse of view. The coastman wants trees, and rocks, and mountain gorges, and fields. Change—an entirety of change—is demanded for real recreation. The preacher wants to go where nobody will know he's a preacher—where he will forget it himself—where there are no sermons to write—where nobody will ask him to officiate—where no books are, and where he can doff his clerical cloth and style, and imagine himself a common mortal like the rest of us. The lawyer wants to go where they never had a court, and where a law book never was seen, an argument never dreamed of, a case never conceived. A doctor wants to go where everybody is healthy, or, if that be impossible, he wants to hide his profession from view and forget pills, squills, ills and bills. The editor wants to go where no news can possibly reach him, and where newspapers never come. He wants to leave his thoughts of his business among his scissors and pens and paste pots. He wants to know nothing of the things that erst have worried him. The banker wants to go where they can't talk loans, nor discounts, nor exchange, nor per cents. The speculator wants to neither see nor hear about the quotations on the street, but to enjoy the blissful pretence that he never dabbled in stocks. The merchant wants to leave every iota of his business thinking behind, and avoid the marts of trade. The mechanic wants to avoid the hum of machinery and make believe he doesn't know a spoke-shave from a rolling-pin, nor a hand-saw from a pan-cake flopper. Everybody on a recreative tour, or excursion, or jaunt of any kind, for the sake of recuperating his impaired energies, wants to do everything and be anything except what he does and is at home.

This is recreation. Doing thus, we get what we go for. This is relaxation of the purest type. The greater the change of scene and occupation from that to which we are accustomed, the greater the benefit we will derive from it. We can't throw off our business too completely when upon a recreational errand. The countryman wants to rig himself out in his Sunday garb and pitch into the very vortex of all those excitements of city life that are new and strange to him; and the city bred man wants to don his roughest attire and go among the simpler people of the rural districts. We cannot make the change too marked, for the greater the change the more thorough the relaxation and the more complete the rest.

But what nonsensical ways of getting recreation are in vogue among the larger number of people. The wealth and fashion of the land seek recreation in only an intenser kind of the same dissipation that has worn them out at home. They dine, and wine, and ride, and dance, and dress all winter at home, and then they betake themselves to a fashionable summer resort where they find the same elegance and luxuries they have left at home, only they dine, and wine, and ride, and dress with ten fold greater zeal that at home, only intensifying their dissipation and coming back with a more fagged out air than they went away with. That's no recreation, and they experience no benefit therefrom, but rather harm.

The average inlander who goes to the woods for a period of recreation, does not do the wisest thing. He may change his habits of living, and his manner of dressing, and his daily fare, and his occupation, but he finds not half the recreation he would get from a sojourn for the same period by the roaring sea, where he hourly comes upon a new experience and is filled with a new wonder every day. There is something so marvelous in the recuperative power of absolutely new scenes and experiences that we stand amazed at their hygienic results. Let a man worn out with work and worry in the mountain regions of Pennsylvania, or on the broad plateaus of Western New York, or upon the expanse of plain we find west and south ; let such a man recreate upon the ocean beach for a month; feed on sea-food; buffet the breakers that roll in from the "vasty deep;" breathe the sea air, and take in that broad view of nature to which he is a stranger at home; and he will be re-made under ordinary circumstances, and go home a different being. Look at the city chap in the country. He can't conceal the complete joy that possesses him in the newness of his experience. He's like a colt for friskness, and the homeliest fare is feasting to him. He is remaking himself—rejuvenating himself—and all because his life in the one place is the antipodes of his life in the other. The change cannot be too radical, and he is wise who, upon determining to spend a special period in recreation, seeks it among surroundings not only new and strange, but the very newest and the very strangest to him that he can select.

This is the principle upon which physicians oftentimes order their patients to pack up and get away from home. It isn't because they need a change of air alone, although that's what they call it, for short, but that they may have a change of everything so far as it is possible to secure such an entire change. It is to start up a new set of ideas, exercise a new set of muscles, bring into play a new set of nerves, and give the worn out ideas, and the tired out muscles, and the shattered out nerves, a good rest. Change of air is good; but no better than a change of food, or water, or exercise, or thinking, or experiencing, or associating. Bring a new toy to a sick child, and it will smile its pleasure through its pain. "We are but children of a larger growth." When we are ailing, and whining, and fretting in that worst of all prisons, a sick room, how eagerly we receive a friend we have not seen for some time, and how it often invigorates and sometimes even cures us. We get tired of, disgusted, with, utterly ennuied over, old and hum-drum things and every day people. We hate the very furniture about us, the paper on the wall, the color or figure of the counterpane, the sight of things out of our window, and even the members of our own family become a sort of endurable necessity. Take us out and away from the old associations, and the old faces, and the old and familiar panorama of every day life, and put us among new surroundings, and we are better at once. This is what the doctors mean by "change of air" and "change of climate" and all that stereotyped sort of doctor talk. They know what human nature is. It wouldn't do for them to say you are only tired of your present environment and need a little shaking up mentally and physically, for you'd pooh-pooh that right back in their faces, and wouldn't budge an inch, lest you be taken for a case of hypo. They appeal to something tangible, and "air" and "climate" and "water" and all that are, as everybody admits, big factors in the popular hygiene. Maybe we've let a cat out of a bag, but we beg forgiveness of our friends, the doctors. There are tricks and tricks, and these tricks are excusable.

The pith of what has been here said is just this: It's come to be decidely hot weather, and we are beginning to bethink ourselves what we shall do to get rid of the bad feelings that annually come over us at this time of the year, owing to our inability to be comfortable, and to its sure sequence, a conclusion that we're in a bad way and must go somewhere. Of course going anywhere is better than staying at home, but it's tenfold better to sit down and think of all the places to which it is possible for us to go, and from among these to choose that which will give us, during our vacation, the greatest possible change from our present mode of living. We cannot be too radical in this unless it be to hit on extremes of temperature. We don't want to rush from tropics to poles, nor *vice versa.* We are supposed to be sane if not sound. But we want a complete change in what we do, and think, and see, and feel, and associate with, as far as practicable. The conventional summering, so called, is worse than sham, it is suicidal. This going from home to re-create but really to disintegrate—going from feasting to gorging—going from occasional to continual dissipation—going from dressing to agonized appareling—going here and there because it is fashionable to do so during certain months of the year, without reference to anything except what "our set" may say if other than the conventional "resort" is visited—this is but an index of mental weakness that calls for our pity, but not for our commiseration. The more we see of the results of this alleged recreation, the more inclined are we to actually respect those weak mortals who coop themselves up in the rearmost apartments of their city dwellings, lock their front doors, close their blinds, and give out to their little world of society that they are gone abroad. It's more rational than to go where they don't recreate, even if they must submit to self imprisonment to prevent self immolation. If good sense comes into exercise in the economy of life at all, it takes a very prominent part in deciding the question, where shall we recreate, and how ?

